# Intestinal tuberculosis mimicking Crohn’s disease with ascending colon sinus tract: a case report

**DOI:** 10.3389/fmed.2026.1777309

**Published:** 2026-03-06

**Authors:** Yiting Lin, Xi Hu

**Affiliations:** Department of Gastroenterology, First Affiliated Hospital of Shantou University Medical College, Shantou, China

**Keywords:** antitubercular therapy, case report, Crohn’s disease, intestinal tuberculosis, sinus tract

## Abstract

**Background:**

Intestinal tuberculosis (ITB) complicated by sinus tract is clinically characterized by nonspecific symptoms. This condition necessitates multidisciplinary collaboration, and a multidimensional differential diagnostic approach integrating clinical manifestations, imaging findings, and pathological examination is imperative for confirmation.

**Case summary:**

A 26-year-old female presented with a 1-month history of recurrent right lower abdominal pain. She had previously been misdiagnosed with appendicitis and Crohn’s disease (CD). Through a comprehensive diagnostic workup including small intestine computed tomography enterography (CTE), purified protein derivative (PPD) test, colonoscopy, and pathological assessment, the patient was diagnosed with ITB complicated by a sinus tract of the ascending colon. Quadruple anti-tuberculosis therapy was initiated. Notably, the fistula achieved complete closure 6 months post-treatment.

**Conclusion:**

This case highlights the critical importance of multidisciplinary diagnosis and treatment in managing ITB. It provides valuable reference material for the clinical diagnosis of such rare complications, thereby contributing to improved clinical decision-making in similar cases.

## Introduction

Tuberculosis (TB) remains a global public health burden. Based on TB incidence data from 173 countries and territories spanning 2010–2019, the projected average global TB incidence in 2030 is 91.581 per 100,000 population (1). In China, the proportion of extrapulmonary tuberculosis (EPTB) among all TB cases was 21.3% (2).

National cross-sectional studies conducted in China between 2020 and 2021 investigated the proportion of Intestinal tuberculosis (ITB) among all EPTB cases was nearly 7% (2, 3). The clinical manifestations of ITB are highly nonspecific. Abdominal symptoms are the most common, with abdominal pain, abdominal distension, nausea, vomiting, diarrhea, and hematochezia being reported in descending order of frequency. Physical signs often include abdominal tenderness, ascites, abdominal masses, jaundice, hepatomegaly, and lymphadenopathy (4, 5). Infection with *Mycobacterium tuberculosis* triggers elicits a granulomatous inflammation (6) with caseous necrosis (7) within the intestinal wall. As a result of this pathological process, ITB can give rise to a spectrum of rare enteric fistulous complications, such as umbilicointestinal, intestinovesical, enteroenteric, and enterocutaneous fistulas (8–10).

ITB follows a prolonged diagnostic trajectory and a frequent diagnostic odyssey across multiple care settings. Non-specific clinical manifestations frequently result in misdiagnosis with malignancies, other infectious disorders, and inflammatory bowel diseases, and contribute to delayed definitive.diagnosis (11). Crohn’s disease (CD) and ITB share common symptoms such as abdominal pain, diarrhea and ascites. Accurate differentiation between ITB and CD holds paramount clinical relevance, as these two entities demand entirely disparate therapeutic paradigms. While anti-tubercular chemotherapy (ATT) constitutes the cornerstone of ITB management, CD mandates long-term immunosuppressive therapy. Inaccurate or postponed diagnosis elicits grave clinical complications, including the progression and extrapulmonary dissemination of occult tuberculosis, which markedly exacerbate patient prognosis and clinical outcomes (12, 13). Microbiological examination for *M. tuberculosis* culture is the gold standard for diagnosing ITB (5). Pathological changes in CD and ITB often occur in the submucosal layer of the intestinal wall. Due to mucosal swelling, endoscopic biopsy samples are usually small and superficial, resulting in low rates of positive findings. At the moment, the key to diagnosing ITB involves a combination of clinical, endoscopic, radiological, and pathological aspects.

The patient in our case report was initially misdiagnosed with appendicitis (with appendectomy) and CD at an outside hospital, and later confirmed to have ITB with sinus tract formation at our center. This report presents the radiological, histopathological, and endoscopic characteristics of ITB, and demonstrates that early differential diagnosis and timely intervention can prevent progression to severe complication, emphasizing the importance of multidisciplinary collaboration in the definitive diagnosis of ITB.

### Timeline

One month prior to admission: CT imaging revealed suspected appendicitis with pericecal exudation. The patient was diagnosed with acute suppurative appendicitis with periappendicitis and underwent laparoscopic appendectomy plus oral antibiotics, but experienced recurrent right lower abdominal pain.

Two weeks prior to admission: Colonoscopy detected multiple ulcers in the terminal ileum/ileocecal region, and pathology showed chronic active inflammation with local granulomas. A diagnosis of Crohn’s disease was established, and oral mesalazine (0.5 g twice daily) was initiated. Symptoms improved but recurred repeatedly.

Final diagnosis: CT enterography (CTE) identified a suspected sinus tract; PPD/IGRA testing was positive, while pathology showed no granulomas and negative acid-fast staining. The diagnosis was revised to intestinal tuberculosis with sinus tract. Anti-TB quadruple therapy was started, and the patient remained free of abdominal pain or fever.

**Figure 1:**
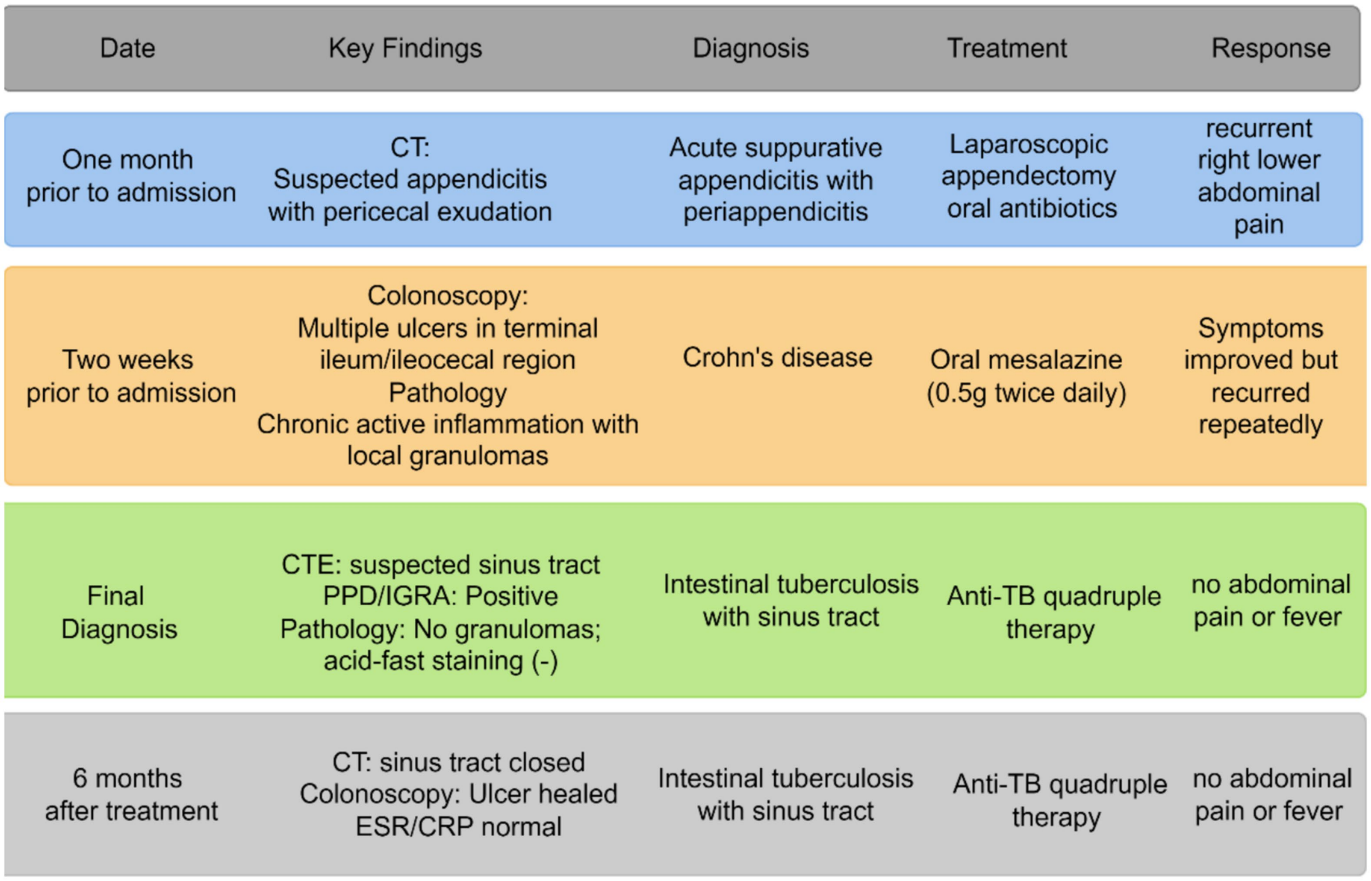
Diagnostic and treatment timeline for a 26-year-old female patient with recurrent right lower abdominal pain.

Six months after treatment: Follow-up CT showed closure of the sinus tract, colonoscopy confirmed ulcer healing, and ESR/CRP levels were normal. The patient continued anti-TB quadruple therapy and remained asymptomatic (See Figure 1).

### Patient information

A 26-year-old woman experienced unexplained pain in her lower right abdomen for over a month, along with abdominal swelling that spread to her right waist and shoulder.

One month prior to admission, an unenhanced abdominopelvic computed tomography (CT) scan at a local hospital indicated suspected appendicitis accompanied by pericecal fluid collection. The patient underwent laparoscopic appendectomy, with a postoperative pathological diagnosis of acute suppurative appendicitis complicated by periappendicitis. She was discharged with oral antibiotics but remained symptomatic with recurrent right lower quadrant abdominal pain.

Two weeks prior to admission, contrast-enhanced abdominopelvic CT identified inflammatory alterations in the ileocecal area. Subsequent colonoscopy revealed multiple ulcerations affecting the terminal ileum, ileocecal region, and ascending colon. Histopathologic analysis of biopsy specimens from the terminal ileum confirmed chronic active inflammation accompanied by focal granuloma formation. Given these findings, the local hospital suspected Crohn’s disease (CD) and initiated oral mesalazine therapy (0.5 g twice daily). Although the patient’s symptoms improved moderately, they relapsed repeatedly, necessitating referral to our institution for further evaluation and targeted treatment. Further management.

### Physical examination

Regular cardiac rhythm. Clear breath sounds bilaterally in the lungs. Soft abdomen. Tenderness was noted in the right lower quadrant without rebound tenderness; no tenderness or rebound tenderness was detected in other abdominal regions.

### Diagnostic assessment

On the day of admission, unenhanced chest CT showed no signs of pulmonary tuberculosis.

On the second day of admission, the tuberculosis-specific interferon-gamma release assay (IGRA) was positive. Procalcitonin was 0.07 ng/mL, C-reactive protein (CRP) was 5.40 mg/L, and erythrocyte sedimentation rate (ESR) was 7 mm/h. Complete blood count showed a white blood cell count of 7.49 × 10^9^/L (neutrophils 66.30%, lymphocytes 24.30%) and a red blood cell count of 5.25 × 10^12^/L, with normal hemoglobin (145 g/L) and platelet count. Serum protein electrophoresis revealed an albumin level of 54.60% and a gamma-globulin level of 19.90%. Immunoglobulin testing demonstrated elevated IgA (4.25 g/L) with normal IgM, IgG, complement C3 and C4. Anti-double-stranded DNA antibody, anti-*β*₂-glycoprotein I antibodies (IgG/IgM), and anti-cardiolipin antibodies (IgG/IgM) were within normal limits. The vasculitis panel (p-ANCA, c-ANCA, MPO-Ab, PR3-Ab) was negative. Quantitative cytomegalovirus and Epstein–Barr virus DNA were undetectable (<4.0 × 10^2^ copies/mL). Tumor markers, coagulation parameters, D-dimer, and routine infectious serology were unremarkable.

On the third day of admission, the PPD test was strongly positive (++++).

**Figure 2:**
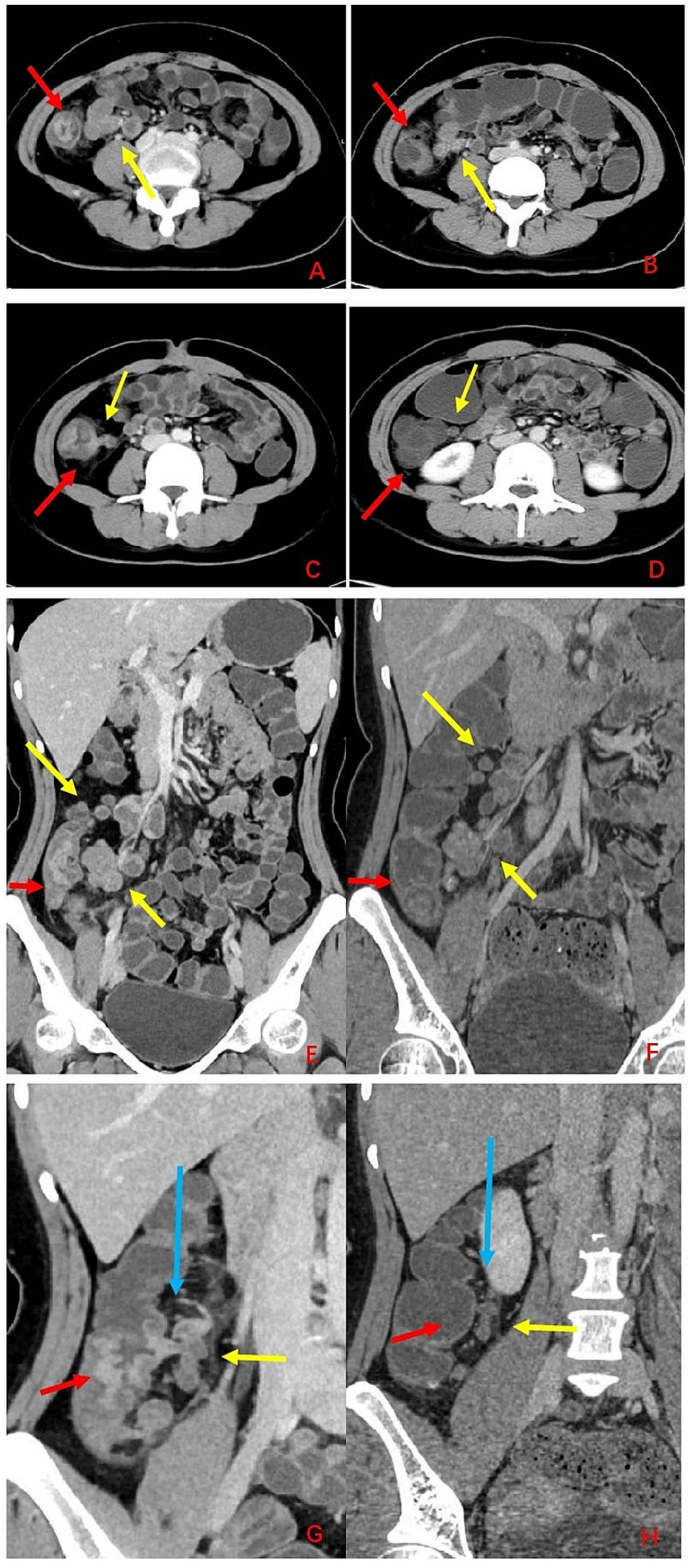
CT imaging findings before and after treatment. **(A,C,E,G)** Pre-treatment CT images. **(B,D,F,H)** Post-treatment CT images. Red arrows indicate lesions; yellow arrows indicate lymph nodes; blue arrows indicate sinus tract.

On the same day, small bowel computed tomography enterography (CTE) revealed asymmetric wall thickening and heterogeneous enhancement involving the ileocecal region, terminal ileum, appendix, and ascending colon. Notably, a sinus tract was suspected to arise from the necrotic intestinal mucosa and communicate with regional mesenteric lymph nodes located medial to the ascending colon (Figure 2). Subsequent gastroscopy demonstrated chronic superficial gastritis.

**Figure 3:**
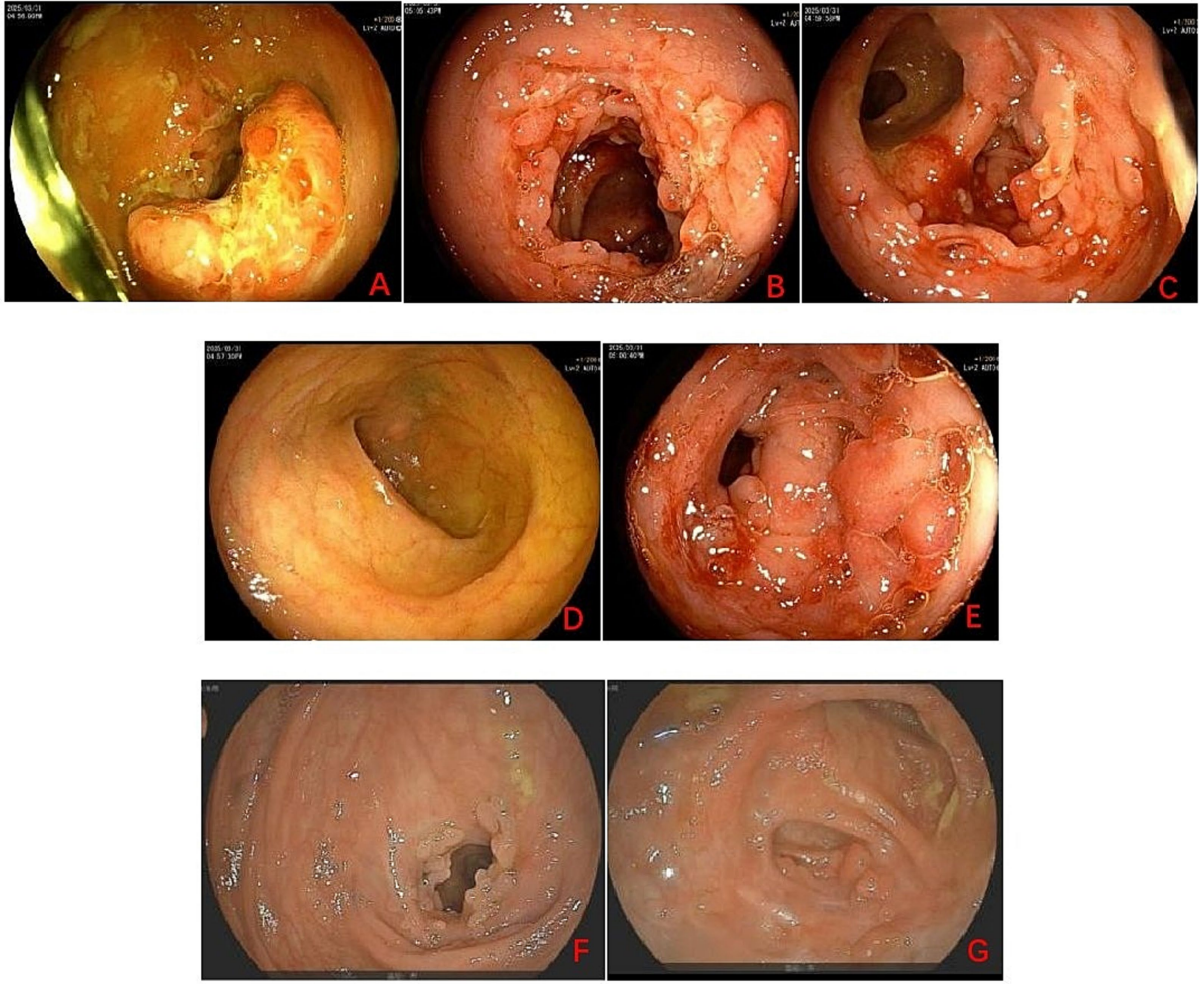
Colonoscopy findings before and after treatment. **(A)** Hepatic flexure. **(B)** ascending colon. **(C)** Ileocecal region. **(D)** Terminal ileum. **(E)** Cecum. Multiple ulcers were observed from the ascending colon to the ileocecal region, accompanied by intestinal stenosis. **(F,G)** Colonoscopic re-examination showed healing of the ulcers.

**Figure 4:**
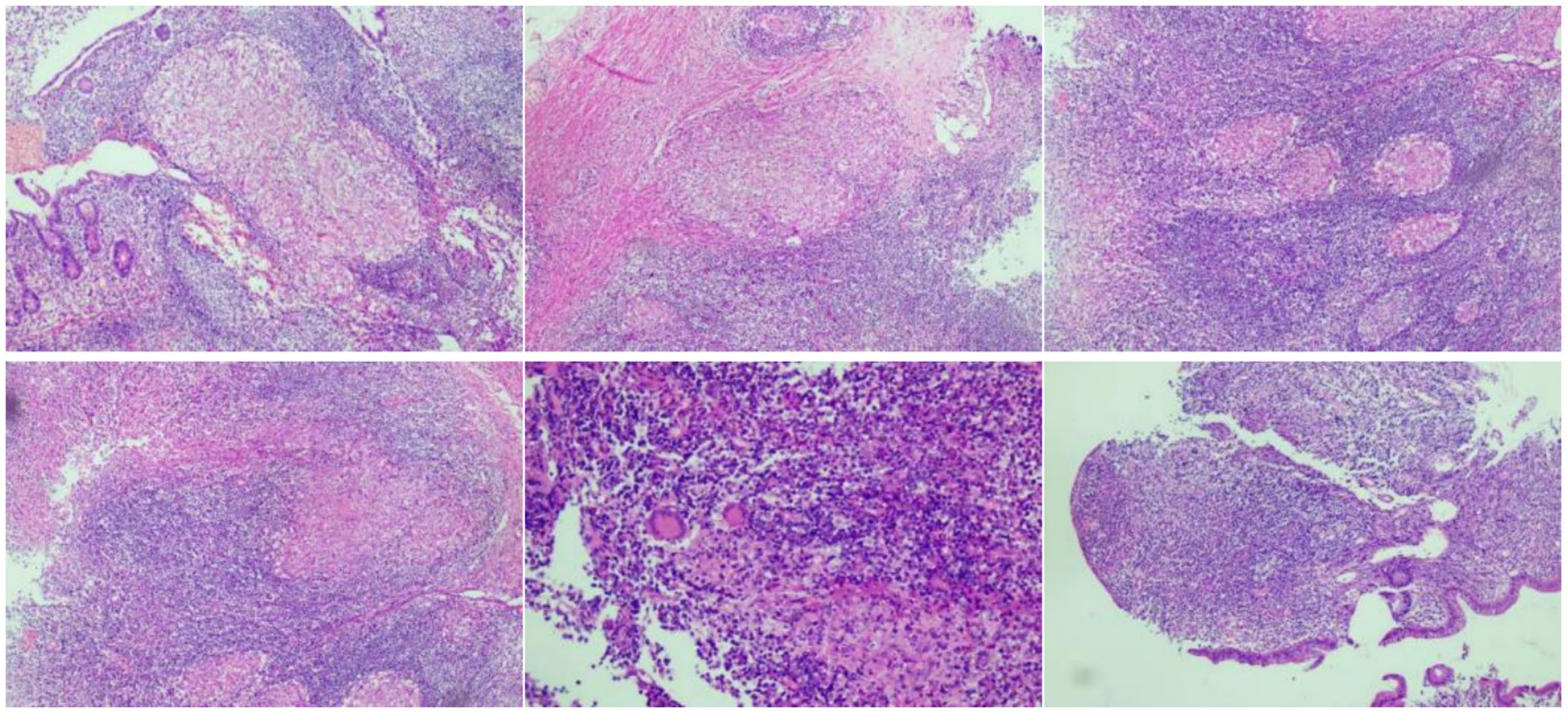
Pathological findings of colon biopsies.

Additionally, colonoscopy performed at our hospital revealed multiple ulcers extending from the ascending colon to the ileocecal region, accompanied by intestinal stenosis, with CD and ITB listed as differential diagnoses (Figure 3). Histopathological examination of biopsy specimens obtained from the rectum, ascending colon, transverse colon, and cecum could not exclude ITB (Figure 4).

Pathological examination of colon mucosa revealed decreased crypt density, irregular glandular architecture accompanied by cryptitis and crypt abscesses. The lamina propria showed massive infiltration of lymphocytes and plasma cells, along with fibrous hyperplasia, inflammatory granulation tissue, and a small amount of necrotic tissue. Special staining (acid-fast staining, periodic acid-Schiff staining, Gomori methenamine silver staining, and Alcian blue staining) yielded negative results.

(A–B) Multiple mesenteric lymph nodes showed a reduction in size and decreased enhancement, and lesions in the ileocecal region, cecum, and ascending colon exhibited a decrease in size and diminished enhancement. (C–D) The original sinus tract between the intestine and adjacent mesenteric lymph nodes showed significant improvement/closure, and the enlarged lymph nodes underwent corresponding size reduction. (E–F) Multiple mesenteric lymph nodes presented a reduction in size and decreased enhancement, and lesions in the ileocecal region, cecum, and ascending colon demonstrated a decrease in size and diminished enhancement. (G–H) The original sinus tract between the intestine and adjacent mesenteric lymph nodes showed significant improvement/closure, and the enlarged lymph nodes were reduced in size.

The patient was ultimately diagnosed with ITB complicated by sinus tract formation in the ascending colon, which arose from necrotic intestinal mucosa and communicated with adjacent mesenteric lymph nodes. The diagnosis of ITB was established based on the following key findings: CTE demonstrated circumferential wall thickening extending from the ascending colon to the cecum, with a sinus tract forming between the intestinal wall and adjacent mesenteric lymph nodes. Colonoscopy identified annular ulcers and proliferative lesions in the ascending colon, accompanied by persistent patency of the ileocecal valve. Histopathological examination revealed epithelioid granulomas with multinucleated giant cells, a characteristic feature suggestive of mycobacterial infection. Notably, no active tuberculous lesions were detected on chest computed tomography (CT); however, both the PPD test and IGRA for tuberculosis yielded positive results.

### Interventions

Quadruple anti-tuberculosis therapy was administered: Isoniazid 0.3 g once daily, Rifampicin 0.45 g once daily, Pyrazinamide 1.5 g once daily, and Ethambutol 0.75 g once daily.

The patient also received nutritional support treatment concurrently.

### Follow-up and outcomes

Three months after treatment, the patient had no abdominal pain, maintained normal ability in daily activities and work, and the quality of life was significantly improved compared with that before treatment. Laboratory tests (erythrocyte sedimentation rate, C-reactive protein were normal). CTE scan demonstrated closure of the fistula (Figure 2) and colonoscopic re-examination showed healing of the ulcer with no signs of recurrence (Figure 3). After fistula closure at 3 months, the patient had no abdominal pain, fever, and maintained normal bowel movements; complete healing at 6 months was confirmed by negative inflammatory markers (CRP, ESR).

## Discussion

This case report describes ITB complicated by sinus tract formation in a patient with a prolonged diagnostic course marked by clinical mimicry of CD. It highlights the critical value of multidisciplinary diagnosis and demonstrates that successful clinical outcomes can be achieved with non-surgical anti-tuberculous therapy alone. Sinus tract formation is an extremely rare complication of ITB. Pathologically, it refers to a narrow, blind-ended tract lined with granulation tissue, with one opening into the intestinal lumen and the other terminating in regional mesenteric lymph nodes. In this case, a multidisciplinary approach involving the Gastroenterology Department, Endoscopy Center, and Radiology Department was adopted. A definitive diagnosis of ITB complicated by sinus tract formation was established through the combined evaluation of colonoscopy, histopathological examination, and CTE, which significantly improved diagnostic accuracy and avoided misdiagnosis. As a single-case report, our findings may be subject to individual variation, and the generalizability of the results is limited. The current follow-up period is 6 months; thus, an extended follow-up duration is warranted to further evaluate the long-term prognosis of this condition.

A retrospective study conducted at Asan Medical Center, a tertiary referral hospital in Seoul, South Korea, between 1996 and 2014, investigated the misdiagnosis rates between CD and ITB (14). The results showed that among 2,760 patients diagnosed with CD, 494 (17.9%) were initially misdiagnosed with ITB, while among 772 patients with ITB, 83 (10.8%) were initially misdiagnosed with CD. In the TB cohort, acid-fast bacilli were detected in 56.3% of cases, while caseating granulomas were identified in 62.1% of the pathological specimens. Clinical manifestations of ITB are non-specific, and histopathological findings may be misleading. Anal fistula features are highly similar between ITB and CD, and key diagnostic markers for ITB show suboptimal detection rates (acid-fast bacilli: 56.3%; caseating granulomas: 62.1%) (15). Thus, when either ITB or CD is suspected on colonoscopy, biopsy specimen culture should be performed alongside histopathological evaluation for granulomas (16). Accurate differential diagnosis between CD and ITB is critically important because misclassification may lead to the administration of immunomodulators, systemic corticosteroids, or biologic agents, which can exacerbate undiagnosed tuberculosis. In a previous case report (17), a patient misdiagnosed with CD and treated with anti-TNF agents following indeterminate latent tuberculosis screening subsequently developed septic shock and intestinal perforation due to disseminated tuberculosis. Accordingly, this case report integrated clinical symptoms, radiological features, endoscopic findings, histopathological results, and multidisciplinary consensus (gastroenterology, endoscopy, and radiology departments) to establish a definitive diagnosis of ITB.

ATT must adhere to the four fundamental principles: early initiation, combination therapy, adequate dosage, and full course of treatment. A clinically effective treatment regimen for pulmonary TB is a 6-month course of combination chemotherapy using isoniazid, rifampicin, ethambutol, and pyrazinamide. Notably, the fundamental principles of pulmonary TB treatment have also been successfully applied to extrapulmonary TB (18, 19). In this case report, after 3 months of anti-infective treatment, endoscopy and imaging showed that the original lesion had completely healed. After fistula closure at 3 months, the patient had no abdominal pain, fever, and maintained normal bowel movements; complete healing at 6 months was confirmed by negative inflammatory markers (CRP, ESR).

## Conclusion

This case demonstrates the diagnostic process of sinus tract formation caused by ITB and emphasizes the crucial role of multidisciplinary diagnosis in improving diagnostic accuracy. Relevant imaging data are provided to raise clinical awareness of ITB and serve as materials for differential diagnosis with other diseases.

## Data Availability

The original contributions presented in the study are included in the article/supplementary material, further inquiries can be directed to the corresponding author.
